# Supplementary material for: Surface Microbiome Profiling of Dental Elevators Using Third‐Generation Sequencing: Implications for Infection Control in Dental Practice
Source: Microbiologyopen. 2025 Dec 8;14(6):e70178. doi: 10.1002/mbo3.70178 (PMC12685768; doi:10.1002/mbo3.70178)
Supplement: Supplementary file 1 — customer_backup. [file MBO3-14-e70178-s001.zip › customer_backup/customer_backup/function_analysis/BugBase/treat/otu_contributions/family/treat.family.Potentially_Pathogenic.pdf]

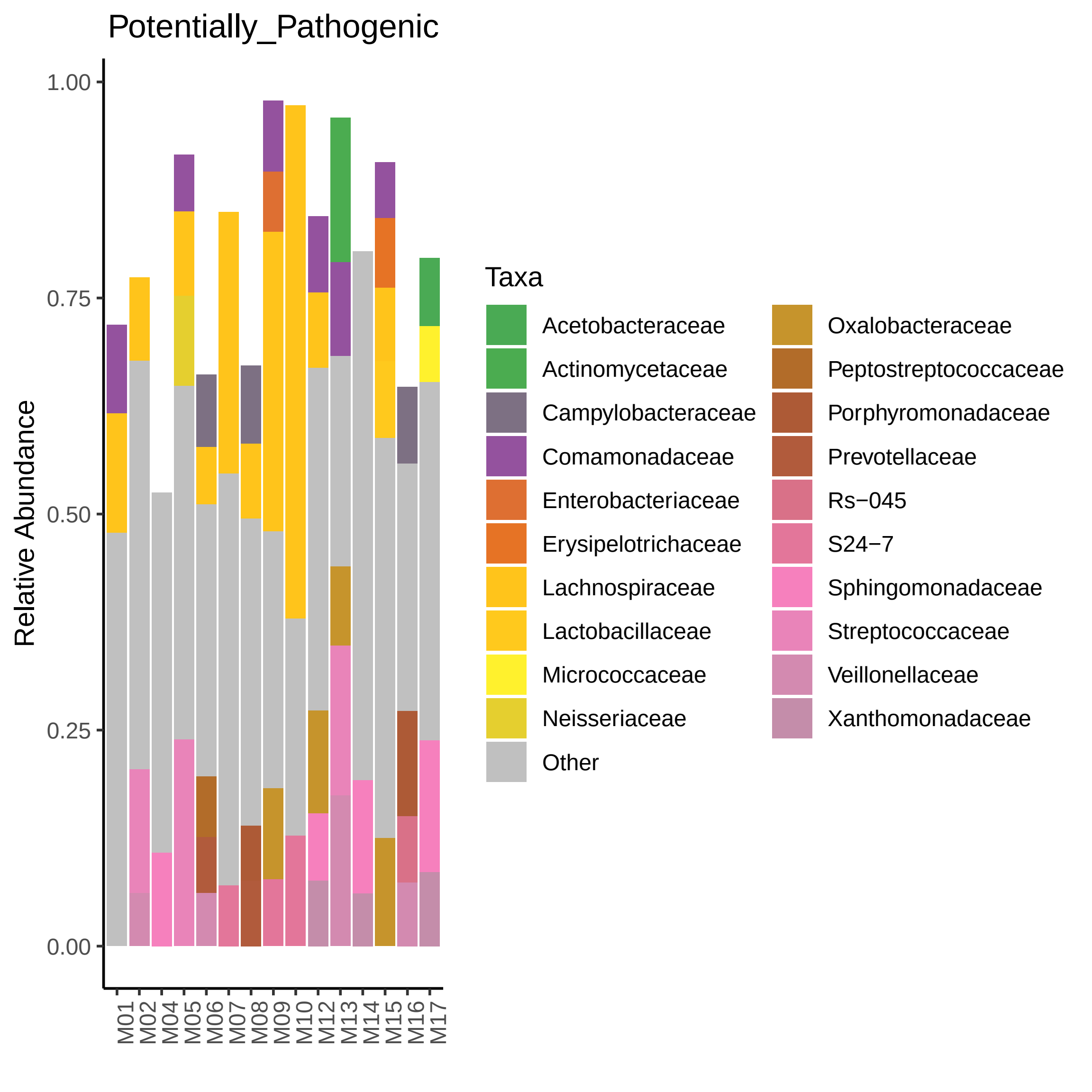
Potentially_Pathogenic
Relative Abundance
1.00
0.75
0.50
0.25
0.00
M01
M02
M04
M05
M06
M07
M08
M09
M10
M12
M13
M14
M15
M16
M17
Taxa
Acetobacteraceae
Actinomycetaceae
Campylobacteraceae
Comamonadaceae
Enterobacteriaceae
Erysipelotrichaceae
Lachnospiraceae
Lactobacillaceae
Micrococcaceae
Neisseriaceae
Other
Oxalobacteraceae
Peptostreptococcaceae
Porphyromonadaceae
Prevotellaceae
Rs−045
S24−7
Sphingomonadaceae
Streptococcaceae
Veillonellaceae
Xanthomonadaceae
